# Graphene Oxide Decorated Nanometal-Poly(Anilino-Dodecylbenzene Sulfonic Acid) for Application in High Performance Supercapacitors

**DOI:** 10.3390/mi10020115

**Published:** 2019-02-11

**Authors:** Nomxolisi R. Dywili, Afroditi Ntziouni, Chinwe Ikpo, Miranda Ndipingwi, Ntuthuko W. Hlongwa, Anne L. D. Yonkeu, Milua Masikini, Konstantinos Kordatos, Emmanuel I. Iwuoha

**Affiliations:** 1 SensorLab, Department of Chemistry, University of the Western Cape, Private Bag X17, Bellville, Cape Town 7535, South Africa; cikpo@uwc.ac.za (C.I.); 3318577@myuwc.ac.za (M.N.); 2962477@myuwc.ac.za (N.W.H.); 3116018@myuwc.ac.za (A.L.D.Y.); mmasikini@uwc.ac.za (M.M.); 2 School of Chemical Engineering, Section I: Chemical Sciences, Lab of Inorganic and Analytical Chemistry, National Technical University of Athens, 9 Heroon Polytechniou Str., 15773 Athens, Greece; ntziouni@mail.ntua.gr

**Keywords:** supercapacitors, graphene oxide, metal nanoparticles, dodecylbenzene sulfonic acid (DBSA) doped polyaniline, capacitance

## Abstract

Graphene oxide (GO) decorated with silver (Ag), copper (Cu) or platinum (Pt) nanoparticles that are anchored on dodecylbenzene sulfonic acid (DBSA)-doped polyaniline (PANI) were prepared by a simple one-step method and applied as novel materials for high performance supercapacitors. High-resolution transmission electron microscopy (HRTEM) and high-resolution scanning electron microscopy (HRSEM) analyses revealed that a metal-decorated polymer matrix is embedded within the GO sheet. This caused the M/DBSA–PANI (M = Ag, Cu or Pt) particles to adsorb on the surface of the GO sheets, appearing as aggregated dark regions in the HRSEM images. The Fourier transform infrared (FTIR) spectroscopy studies revealed that GO was successfully produced and decorated with Ag, Cu or Pt nanoparticles anchored on DBSA–PANI. This was confirmed by the appearance of the GO signature epoxy C–O vibration band at 1040 cm^−1^ (which decreased upon the introduction of metal nanoparticle) and the PANI characteristic N–H stretching vibration band at 3144 cm^−1^ present only in the GO/M/DBSA–PANI systems. The composites were tested for their suitability as supercapacitor materials; and specific capacitance values of 206.4, 192.8 and 227.2 F·g^−1^ were determined for GO/Ag/DBSA–PANI, GO/Cu/DBSA–PANI and GO/Pt/DBSA–PANI, respectively. The GO/Pt/DBSA–PANI electrode exhibited the best specific capacitance value of the three electrodes and also had twice the specific capacitance value reported for Graphene/MnO_2_//ACN (113.5 F·g^−1^). This makes GO/Pt/DBSA–PANI a very promising organic supercapacitor material.

## 1. Introduction

The world has been facing global warming and energy problems with Earth’s natural resources depleting at a very rapid rate. In 2007, the International Energy Agency published the World Energy Outlook which estimated that by 2030 there will be 55% more energy demand as compared to today [1]. Global economic development and prosperity have been built on cheap and abundant fossil fuels with petroleum standing at 39%, natural gas at 24% and coal at 23%, but there is a limited amount of fossil fuels and they are non-renewable [2]. Estimates state that there is a 2% annual growth in global oil demand along with a natural decline in production from existing reserves [3]. Therefore, there is need to invest in alternative sources of energy both in terms of energy conversion, as well as storage devices such as electrostatic capacitors, electrochemical capacitors (supercapacitors), batteries, and fuel cells. Metal oxides and transition metal oxides have been utilized for the development and utilization of smart materials in applications such as gas sensors [4], energy storage smart materials [5,6] and advanced energy conversion devices. Perovskite solar cells (PSCs) have attracted a great deal of attention in the photovoltaic cell field of study, due to their high photo-to-electric power conversion efficiency (PCE) and low cost. The high PCE is due to the high and excellent physical properties of organic–inorganic hybrid perovskite materials. These include a long charge diffusion length and high absorption coefficient in the visible range [7]. Titanium oxide (TiO_2_) nanostructures are excellent anode materials for sodium ion batteries due to their inherent safety, low cost and structural stability. When tested as a binder and conducting additive-free electrodes in sodium cells, TiO_2_ nanotubular arrays, obtained from simple anodic oxidation, exhibited different electrochemical responses which then render TiO_2_ as a good anodic material [8]. Perreault et al., developed a spray-dried mesoporous mixed Cu–Ni oxide and graphene nanocomposite microspheres for high power and durable Li-ion battery anodes. They exhibited unprecedented electrochemical behavior such as high reversible specific capacity, excellent coulombic efficiency and long-term stability at high current density that are very remarkable when compared to most traditional metal oxides and nanocomposites prepared by conventional techniques [9].

Supercapacitors are electrical energy storage devices that store and discharge energy at the electrochemical interface and utilize the three-electrode system, i.e. working electrode, counter electrode and reference electrode [10,11]. Supercapacitors have very high capacities and low resistance and are able to store energy at relatively higher rates, when compared to other energy storage devices. This is due to the mechanism of energy storage which involves a simple charge separation at the interface between the electrode and electrolyte [12]. Supercapacitors have attracted attention due to their unique and wide potential in a variety of applications such as electric vehicles, power back-up in mobile phones, digital cameras, radio tuners, laptops, etc. and they find application as power back-ups for uninterruptible power system (UPS) applications and other high-power apparatus [13,14]. Supercapacitors are classified into three main general categories: (i) electric double-layer capacitors (EDLC), (ii) Faradaic pseudocapacitors and (iii) hybrid capacitors [2]. Electric double-layer capacitors (EDLC) are known to utilize carbon-based materials such as graphene oxide, carbon black, activated carbon, etc. Faradaic pseudocapacitors are known to utilize metal oxide and/or conducting polymers. Hybrid capacitors are known to utilize carbon materials and metal oxides and/or conducting polymers [15,16]. This work uses graphene oxide (GO), metal nanoparticles (Ag, Cu and Pt) and dodecylbenzene sulfonic acid (DBSA)-doped polyaniline (PANI) as the materials to use in the development of high capacitance supercapacitors. GO has considerable exceptional properties such as mechanical, optical, electronic, electrical, etc. which then render GO as a good candidate for use in energy storage devices [17,18]. Metal nanoparticles have attracted attention due to the performance in electronic, optical, magnetic, and catalytic applications and, recently, these metal nanoparticles have been supported on the surface of GO [19,20,21]. It is expected that small sized and well-dispersed nanoparticles will enhance activity and selectivity for catalytic applications [22,23]. Conducting polymers have been used to maintain the need for high electrical conductivity of materials for a myriad of applications including energy-related devices [24,25]. There has been much interest in PANI-based materials because of their low cost and easy synthesis [26]. Since PANI is an excellent organic conductor with good environmental stability, good electronic and optical properties, is highly stable in air, and is soluble in several solvents [27], it has been used often to produce materials or composites with carbon materials for supercapacitor electrodes or sensor applications [28]. The combination of the properties of these materials is expected to increase the capacitance of the developed supercapacitors [29]. Furthermore, some organic polymers have been found to be suitable materials for supercapacitor applications, for which the incorporation of cations, such as metal molybdates, leads to increased specific capacitance [30,31,32]. 

Table 1 is a comparison of specific capacitances, energies and powers of different supercapacitor electrode materials reported by researchers over the past years. From the table, it can be deduced that the material used in this work exhibited higher capacitance, energy and power values when compared to graphene-containing supercapacitor material [33]. Metal oxides combined with carbon-based materials and conducting polymers give the best capacitance ranging from 200 to 400 F·g^−1^ and low resistances. They also give highly specific energy and power. From the table, we can observe that manganese oxide combined with e-CMG and tantalum (IV) oxide combined with PANI–PSSA gives higher specific capacitances of 389 and 318.4 F·g^−1^ respectively. Lithium manganese oxide combined with aluminum oxide gives the best specific energy of 864.3 Wh kg^−1^ and titanium oxide combined with carbon nanotubes gives the best specific power of 6428 W kg^−1^.

To the best of our knowledge, no work has been published on GO decorated with Ag, Cu and Pt nanoparticles that are anchored on DBSA–PANI, for application in high performance supercapacitors.

## 2. Materials and Methods

### 2.1. Materials

All chemicals used in the experiments were of analytical grade and were used as purchased without further purification. Graphite (1–2 µm); sodium nitrate, NaNO_3_ (≥99%); sulfuric acid, H_2_SO_4_ (≥98%); potassium permanganate, KMnO_4_ (≥99%); hydrogen peroxide, H_2_O_2_ (≥30%); hydrochloric acid, HCl (≥37%); hexachloroplatinic acid, H_2_PtCl_6_6H_2_O, ACS reagent grade (≥37.50%); Pt wire; silver nitrate AgNO_3_, ACS reagent grade (≥99.0%); sodium hydroxide, NaOH, BioXtra (≥98%) acidimetric, pellets (anhydrous); cetyl trimethylammonium bromide, CTAB (98%); poly(sodium 4-styrenesulfonate), PSS (∼70000) powder; sodium acetate, NaAc; poly(ethylene glycol), PEG (400 powder); ethylene glycol anhydrous, EG (99.8%); acetone (≥99.9%); dodecylbenzenesulfonic acid, DBSA (≥98%); aniline, ACS reagent grade (≥99.5%); ammonium persulfate, ACS reagent grade (≥98.0%); methanol (≥99.9%); and isopropanol (≥99.7%), were all purchased from Sigma-Aldrich (Modderfontein, South Africa).

### 2.2. Instrumentation

The cell system was fabricated using 1 M H_2_SO_4_ solution as the electrolyte and tested for the supercapacitor parameters using the BST8-3 eight-channel battery testing machine. Fourier transform infrared (FTIR) spectra were recorded on a 100 spectrophotometer, Perkin Elmer Fourier Transform Infrared model (USA), operating between 400 and 4000 cm^−1^ in order to characterize the presence of specific features of the materials. The high-resolution scanning electron microscopy (HRSEM) of GO measurements were made with a Ziess Auriga, Hitachi S3000N, Quorum Technology (Lewes, England), operating at 50 kV and high-resolution transmission electron microscopy (HRTEM) measurements were made with Tecnai G2 F20X-Twin MAT Field Emission Transmission Microscopy. FEI (Eindhoven, Netherlands) equipped with an energy-dispersive spectroscopy (EDS) detector was used to study the size and morphology of the samples. Copper grid (Cu) was used as a sample holder for the immobilization of (2 μL) solution of GO, GO/Ag/DBSA–PANI and GO/Pt/DBSA–PANI and a nickel grid for GO/Cu/DBSA–PANI, and the micrographs were recorded at room temperature. Cyclic voltammetry (CV) and electrochemical impedance spectroscopy (EIS) measurements were made with VMP 300 Bio-Logic SAS, Biologic Science Instruments (Seyssinet-Pariset, France), where all cyclic voltammograms and EIS graphs were recorded with a computer interfaced with VMP 300 Bio-Logic SAS using a 10 mL electrochemical cell that has a three-electrode system. The electrodes used were: (1) 0.071 cm^2^ glassy carbon electrode (GCE) as the working electrode, (2) 500 mm × 0.635 mm platinum wire electrode (Pt) from Sigma-Aldrich as the counter electrode, and (3) Ag/AgCl electrode (with a 3 M NaCl salt-bridge) as the reference electrode. Alumina micropolishing pads were obtained from Buehler, LL, USA and were used for polishing the glassy carbon electrode before modification. Galvanostatic charge-discharge measurements were taken with 8-Channels Battery analyzer BST8-3, MTI Corporation (Richmond, VA, USA) using an electrochemical cell and the three-electrode system with a potential sweep rate of 0.9 mV s^−1^.

### 2.3. Experimental

#### 2.3.1. Synthesis of GO

GO was prepared using a modified Hummers method. Specifically, 0.5 g of graphite powder was added to a cold solution of 40 mL of concentrated sulfuric acid (H_2_SO_4_) and 0.375 g of sodium nitrate (NaNO_3_) under vigorous stirring for 1 h in an ice bath. A mass of 2.25 g potassium permanganate (KMnO_4_) was added portion wise to the solution while it was stirring and the mixture remained in the ice bath for a further 2 h to cool the mixture below 10 °C. The mixture took on a green brown color and stirring continued for 5 days in order to ensure complete oxidation of the graphite. After completion of the reaction, 70 mL of dilute aqueous solution of 5% H_2_SO_4_ was added to the mixture to cleave the formed precipitate salts due to oxidation. The mixture was heated and stirred at 98 °C for 1 h. The heating was removed and 2 mL 30% H_2_O_2_ peroxide was added to the mixture (after it was let to cool down to 60 °C). The mixture was stirred for a further 2 h. Subsequently, in order to remove the residues of KMnO_4_ and derivatives such as Mn_2_O_7_, the following procedure was followed. The mixture was centrifuged for 10 min at 4000 rpm, washed with 600 mL aqueous solution of 3% H_2_SO_4_ and 0.5% H_2_O_2_ and then placed in an ultrasonic bath for 10 min. The process was repeated a number of times to get rid of the salts. Then, the mixture was washed and purified with 150 mL of aqueous 3% HCl two to three times by mixing and centrifugation, to eliminate any metal ions. Then, the mixture was washed with distilled water until (average of four washings) the pH increased to the value of 7, and thereby ensuring the removal of any remaining acidic. Finally, the solution was washed with acetone and dried at 60 °C in a vacuum oven for 12 h. After drying, the GO was obtained in the form of a shell, followed by grinding, weighing and collecting the product [38]. 

#### 2.3.2. Synthesis of Graphene Oxide Loaded with Pt, Ag and Cu NPs

The loading of platinum, silver and copper NPs onto GO nanosheets was carried out by electrostatic self-assembly (scheme 1). Initially, GO was functionalized by cetyl trimethylammonium bromide (CTAB), a cationic polyelectrolyte which acts as a surfactant and poly(sodium 4-styrenesulfonate) (PSS) an anionic polyelectrolyte. Thirty milligrams of GO was homogeneously dispersed in 40 mL of an aqueous solution of 1% wt. CTAB using ultra-sonication for 30 min followed by centrifugation to remove the remaining excess CTAB. The functionalized GO was then dispersed in 40 mL of an aqueous solution of 1% wt. PSS by stirring and ultra-sonication for 30 min and the mixture was stored overnight. After 12 h, the excess PSS was removed by centrifugation and the prepared material was subjected to ultrasonic agitation in 40 mL of ethylene glycol (EG) for 30 min. Consequently, 0.2 g of hexachloroplatinic acid (H_2_PtCl_6_6H_2_O) for platinum nanoparticles (scheme 1), silver nitrate (AgNO_3_) for silver nanoparticles and copper acetate Cu(CH_3_COO)_2_ for copper nanoparticles, respectively, were dissolved in the 40 mL dispersion of EG/functionalized GO and the mixture was sonicated for 30 min to form a stable suspension. At this point, 3.6 g of sodium acetate (NaAc) and 1.0 g of poly-ethylene glycol (PEG) were added under continuous stirring for a further 30 min. The suspension was then sealed in a Teflon autoclave of stainless steel (capacity 100 mL) and heated at 200 °C for 12 h followed by natural cooling to room temperature. A black precipitate was obtained by filtration, washed by de-ionized water and acetone and dried in a vacuum oven at 60 °C for 12 h [39].

#### 2.3.3. Synthesis of GO/Pt NPs, GO/Ag NPs and GO/Cu NPs Anchored DBSA-Doped PANI

A mass of 0.5 g GO/Ag NPs, GO/Cu NPs and GO/Pt NPs was sonicated in 400 mL of 1 M DBSA solution prepared in 1 M HCl for 5 h. DBSA, an anionic surfactant, was used as the dopant as well as to disperse GO/Ag NPs, GO/Cu NPs and GO/Pt NPs in the solution. DBSA is an anionic surfactant used to blend polyaniline with the graphene oxide-loaded nanoparticles. Thereafter, 5 mL of double distilled aniline and solution of ammonium persulfate (12.53 g of (NH_4_)_2_S_2_O_8_ in 100 mL of 1 M HCl) were added drop-wise to the previous solution of DBSA for in situ oxidative polymerization of aniline with GO/Ag NPs, GO/Cu NPs and GO/Pt NPs under stirring conditions in an ice bath for 6.5 h (scheme 1). A greenish black precipitate was obtained, which was washed thoroughly with double distilled water and methanol to remove any traces of reactants and PANI oligomers until the filtrate became transparent. Thus, the prepared nanocomposite was dried at 60 °C and stored in a desiccator for further experiments [40]. The materials were named GO/Ag/DBSA/PANI, GO/Cu/DBSA–PANI and GO/Pt/DBSA–PANI, respectively.

### 2.4. Characterization of Electrode Materials

Characterization of the electrode materials was carried out using the following techniques: HRTEM, HRSEM, EDS and FTIR. In summary, elemental compositions of the GO, GO/Ag/DBSA–PANI, GO/Cu/DBSA–PANI, and GO/Pt/DBSA–PANI were quantitatively studied by the EDS. The morphological properties and degree of agglomerations of the nanostructures were studied qualitatively by HRSEM. The structural properties and composition of materials were studied using FTIR. Particle shape, particle size and morphological distribution of GO, GO/Ag/DBSA–PANI, GO/Cu/DBSA–PANI, and GO/Pt/DBSA–PANI were qualitatively studied by HRTEM and HRSEM. The stability and efficiency of the electrode material synthesized and used for supercapacitor electrodes was tested using a potentiostatic-galvanostatic charge-discharge test.

### 2.5. Fabrication of the Electrode Material

#### 2.5.1. Preparation of the Electrode Materials

The materials used in the experiment for fabrication of the electrode consisted of 40 mg of active material, 5 mg of carbon black, 5 mg (3 drops) of isopropanol, and 8 mg of polytetrafluoroethylene (PTFE) binder. The active material consisted of GO/Ag/DBSA–PANI. The carbon black and GO/Ag/DBSA–PANI were mixed together and crushed to ensure that they were correctly mixed. For a given electrode, relevant materials were mixed together in a 10 mL small beaker to form dough. The dough was transferred onto a flat glass plate. A stainless steel/Teflon rod was used to roll the dough into 1 mm-thick flexible thin films. When making the thin film, the dough was rolled many times with the constant addition of three drops of isopropanol to ensure that the material was correctly mixed in the thin film. The thin film was then placed in an oven and was allowed to bake at 80 °C under vacuum. Once the thin film was dry it was then cut into small wafers for the construction of the electrode. The same principle was used for GO/Cu/DBSA–PANI and GO/Pt/DBSA–PANI composites [17].

#### 2.5.2. Construction of Supercapacitor Cell

A single electrode was assembled with three parts electrode material, stainless steel mesh current collector and stainless-steel wire. The electrode was assembled by cutting the stainless-steel mesh current collector into a 1 cm × 4 cm rectangular shape. The collector was then cleaned by shaking it in ethanol, drying it and then weighing. The approximately 1 cm^2^ wafer was placed on the stainless-steel mesh and pressed at a pressure of 20 MPa for 5 min. The electrode was then weighed and the difference in mass was used as the active mass of the electrode (which was 0.021 g for the anode and 0.0190 g for the cathode). The stainless-steel wire was tightly held onto the current collector for external circuit connection and acted as a cathode. The active material was GO/Ag/DBSA–PANI and acted as an anode. The stainless-steel wire and active material were used to make a two-electrode asymmetric supercapacitor cell. The cell system was fabricated using 1 M H_2_SO_4_ solution as the electrolyte and tested for the supercapacitor parameters using the BST8-3 eight-channel battery testing machine. The cell was fabricated by holding together the two single electrodes (cathode and anode) with a porous and electronically non-conductive separator sandwiched between them to form the cell configuration [34]. The same principle used GO/Cu/DBSA–PANI and GO/Pt/DBSA–PANI.

## 3. Results and Discussion

**Figure 1:**
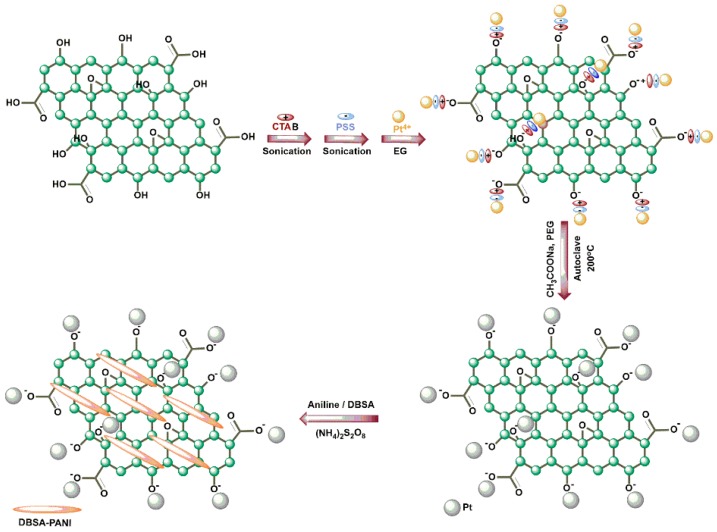
Schematic illustration for the synthesis of a GO/Pt/DBSA–PANI nanocomposite.

The synthesis route for producing GO/Pt/DBSA–PANI nanocomposite is illustrated in Figure 1. A similar experimental process was followed for obtaining GO/Ag/DBSA–PANI and GO/Cu/DBSA–PANI by using the corresponding salts of Ag and Cu as starting materials.

**Figure 2:**
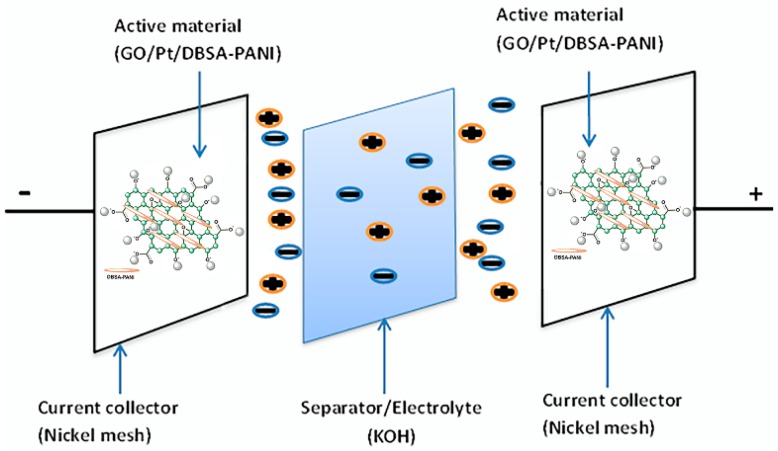
Schematic illustration of the GO/Pt/DBSA–PANI-based supercapacitor cell.

Figure 2 shows the schematic diagram of the supercapacitor cell which consists of an electrolyte (KOH), two electrodes and a separator that electrically separate the electrodes. The active material (GO/Pt/DBSA–PANI) of the electrodes is considered one of the most important components of supercapacitors, as the capacitance depends on the type and properties of the electrode material. 

### 3.1. Morphology Characterization

**Figure 3:**
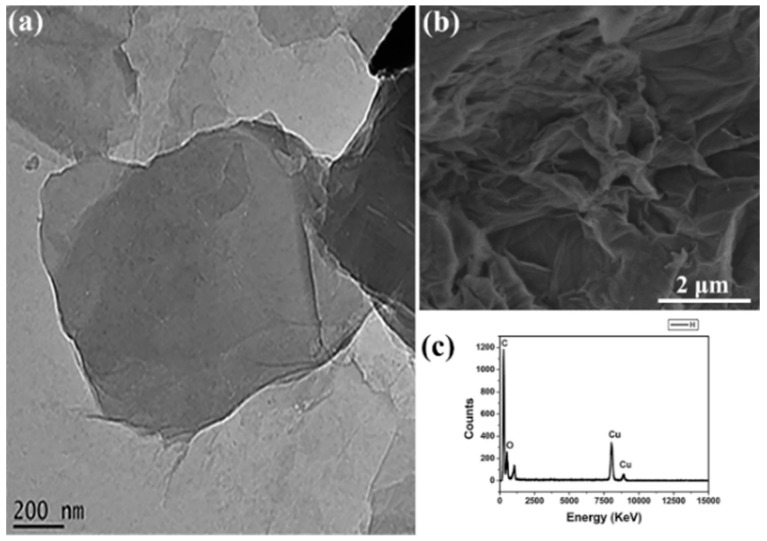
High-resolution transmission electron microscopy (HRTEM) image (**a**), scanning electron microscope (SEM) image (**b**) and energy-dispersive spectroscopy (EDS) spectrum of GO (**c**), respectively.

Figure 3a demonstrates that GO retains a graphene-like lattice substructure which is due to the ultra-sonication of GO [41]. The nanosheets are observed to be flat, light, and transparent and they appear to be larger than 1.5 µm and to be situated on top of the copper grid which is used during HRTEM analysis [42]. The wrinkles and the bends that are observed are due to the abundant defects and functional groups during the oxidation process which takes place over a period of 5 days [43,44]. These nanosheets also appear to be extremely dispersed in water due to the existence of topological features along the overlapping of the nanosheets [45,46,47]. Figure 3b shows the SEM image of GO, highlighting that GO was efficiently exfoliated to form thin wrinkled sheets with porous structures [48,49]. The images also resemble sponge-like structures due to the well-defined and interlinked three-dimensional graphene sheets [50]. The EDS spectrum in Figure 3c shows the elemental composition of GO and confirms that GO was oxidized due to the presence of the GO functional groups i.e. carbon and oxygen [50,51].

**Figure 4:**
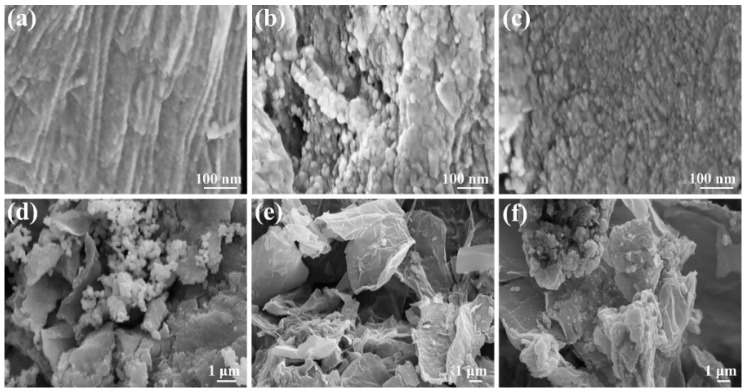
HRSEM images of (**a**) GO–Ag NPs, (**b**) GO–Cu NPs, (**c**) GO–Pt NPs, (**d**) GO/Ag/DBSA–PANI, (**e**) GO/Cu/DBSA–PANI, and (**f**) GO/Pt/DBSA–PANI.

Figure 4a–c shows the HRSEM images of GO–Ag NPs, GO–Cu NPs and GO–Pt NPs, respectively. As observed from the images, when the GO surface was loaded with the nanoparticles, the surface changed from smooth to rough with small particles observed to be situated on the surface of GO. Upon magnification, the nanoparticles appeared to be spread out on the surface of GO and, therefore, this confirms that GO was loaded with the nanoparticles [52,53,54]. Figure 4d–f shows the HRSEM images of GO/Ag/DBSA–PANI, GO/Cu/DBSA–PANI and GO/Pt/DBSA–PANI, respectively. The M/DBSA–PANI (M = Ag, Cu or Pt) nanocomposites are observed as aggregated particles that are adsorbed on the GO surface. The surfaces and the edges are toothed, rough and very much agglomerated [55,56,57,58].

**Figure 5:**
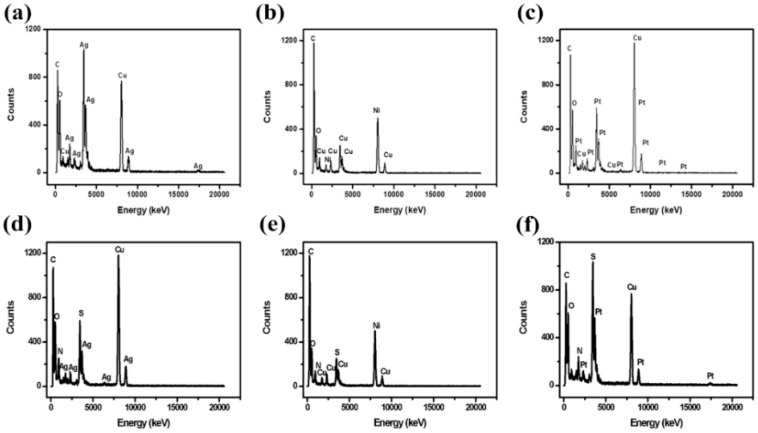
EDS images of (**a**) GO–Ag NPs, (**b**) GO–Cu NPs, (**c**) GO–Pt NPs, (**d**) GO/Ag/DBSA–PANI, (**e**) GO/Cu/DBSA–PANI and (**f**) GO/Pt/DBSA–PANI.

Figure 5a–c shows the EDS spectra of GO/Ag NPs, GO/Cu NPs and GO/Pt NPs, respectively. This confirms the presence of the metal nanoparticles loaded on the surface of GO [59,60,61]. However, the presence of copper is attributed to the copper grid used upon sample preparation for the GO–Ag NPs and GO-Pt NPs, but in the case of GO–Cu NPs, a nickel grid was used so as to observe the presence of Cu NPs. Figure 5d–f shows the EDS spectra of GO/Ag/DBSA–PANI, GO/Cu/DBSA–PANI and GO/Pt/DBSA–PANI and the quantitative analysis result indicates the presence of carbon, sulfur, platinum, silver, and copper in the polymer composites. Also, the result confirms the formation of platinum, silver and copper nanoparticles [62]. The presence of the sulfur is due to DBSA and this confirms that GO was anchored with DBSA–PANI [63].

**Figure 6:**
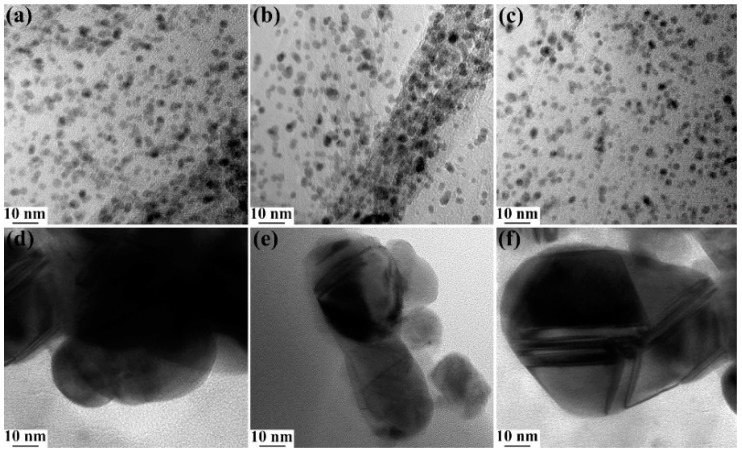
HRTEM images of (**a**) GO-Ag NPs, (**b**) GO-Cu NPs, (**c**) GO-Pt NPs, (**d**) GO/Ag/DBSA–PANI, (**e**) GO/Cu/DBSA–PANI, and (**f**) GO/Pt/DBSA–PANI.

Figure 6a–c shows HRTEM images of GO–Ag NPs, GO–Cu NPs and GO–Pt NPs, respectively. The nanoparticles are small-sized and well-dispersed on the surface of GO with mean particle sizes of 2.6 ± 0.3 nm, 3.5 ± 0.5 nm and 2.3 ± 0.2 nm for Ag, Cu and Pt NPs, respectively. Upon heat treatment, there was no aggregation of the nanoparticles, hence the nanoparticles are small-sized. There is also a strong interaction between the nanoparticle atoms and GO [64]. Figure 6d–f shows the surface structures of GO/Ag/DBSA–PANI, GO/Cu/DBSA–PANI and GO/Pt/DBSA–PANI, respectively. They are observed to be very dark due to the presence of GO in the polymer matrix. DBSA acts as a surfactant and binding agent and assists in binding the polymer and the GO [65]. The single GO sheets may also be embedded into the polymer matrix, which causes the DBSA–PANI particles to become adsorbed on their surfaces and this process then appears as dark surfaces in the HRTEM images of the materials [66]. Agglomerates appear as dark regions in the HRTEM images, thus supporting that single GO sheets are embedded into the polymer matrix and DBSA–PANI particles are adsorbed onto the surface of GO [67,68,69,70].

### 3.2. Molecular Structure Characterization—Fourier Transform Infrared (FTIR)

**Figure 7:**
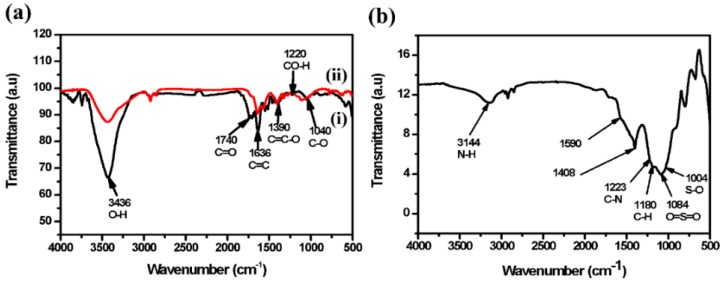
Fourier transform infrared (FTIR) spectra of (**a**) GO (i) and GO/Pt NPs (ii) and (**b**) GO/Pt/DBSA–PANI.

Figure 7a shows the FTIR spectrum of GO/Pt NPs (ii) compared with the GO spectrum (i). GO contains carbon and oxygen functional groups, mainly O–H at 3436 cm^−1^ attributed to hydroxyl and carboxylic acid functionalities, C=O at 1740 cm^−1^, C=C at 1636 cm^−1^, C=C-O at 1390 cm^−1^, CO-H at 1220 cm^−1^ attributed to the functionality of graphene sheets and C–O at 1040 cm^−1^ was related to the vibration of epoxide functionality [71]. The appearance of all these vibrational bands indicates the presence of rich oxygen-containing functionalities in graphene oxide [72]. When the ethylene glycol was introduced in the synthesis process, it reduced the platinum precursor and GO and the band intensities revealed a decrease in the functional groups of the epoxide and carbonyl which indicates the incomplete reduction of GO [73,74]. GO–Ag NPs and GO–Cu NPs behaved the same way. FTIR studies of GO/Pt/DBSA–PANI observed in Figure 7b revealed a broad absorption band at around ~ 3144 cm^−1^, which corresponds to polyaniline N–H stretching vibrations [27,75]. The vibrational bands centred at 1590 and 1408 cm^−1^ can be attributed to the stretching frequencies of quinoid and benzenoid rings of polyaniline, respectively [76]. The band that is produced at 1223 cm^−1^ belongs to the C–N stretching of the secondary amide group [52]. The band at 1180 cm^−1^ is attributed to in-plane bending of the C–H bond, and bands at 1084 and 1004 cm^−1^ are due to the SO_3_^−^ group (O=S=O and S-O stretching) of DBSA [77,78,79]. FTIR studies of GO/Ag/DBSA–PANI and GO/Cu/DBSA–PANI revealed the same pattern. Table 2 shows and confirms the frequencies and their respective bonds found in (a) (ii) GO/Pt NPs and (b) GO/Pt/DBSA–PANI.

### 3.3. Electrochemistry

#### 3.3.1. Cyclic Voltammetry

**Figure 8:**
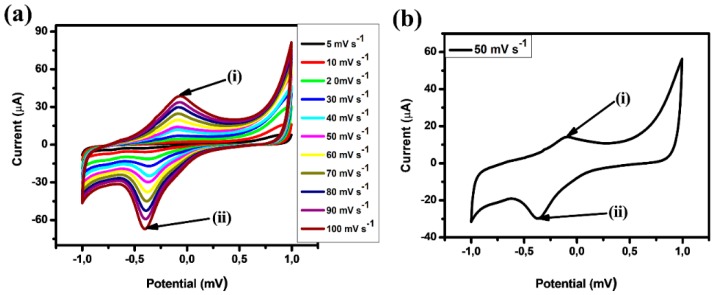
Cyclic voltammetry (CV) behaviour of (**a**) GO at 5–100 mV·s^−1^ and (**b**) GO at 50 mV·s^−1^. The electrochemical behaviour of GO was studied in the potential range of −1.0 V to +1.0 V in 0.1 M KOH.

The electrochemical performances of GO were measured in a symmetrical two-electrode cell in the potential range of −1.0 V to +1.0 V in 1.0 M KOH. Figure 8a exhibits the cyclic voltammogram of GO in which one anodic peak (i) and one cathodic peak (ii) are observed during the process. The anodic peak (i) and cathodic peak (ii) are credited to the electrochemically active oxygen functional groups of reduced planes of GO [34,80,81]. The persistent increase of the peak currents shown with successive potential scans as observed in Figure 8a, indicates that the deposition of GO on glassy carbon electrode (GCE) has been achieved. Figure 8b shows that the deposited glassy carbon electrode (GCE) modified with the material GO displays an anodic peak (i) and cathodic peak (ii), which are attributed to the large number of electrochemically active oxygen-containing groups of GO planes that are very stable [82,83]. The capacitive current covering from −30 to 0 μA in the CV of Figure 7b, indicates that the materials exhibit pseudocapacitance behavior. The current under the curve is slowly increased with the scan rate of CV, which then reveals that voltammetric current is directly proportional to the scan rates of CV [75]. The capacitance, C, can be calculated by the following equations where Q is the positive voltammetric charge in (V); I is the average current in (A) and $\frac{\mathrm{dV}}{\mathrm{dt}}$ is the voltage scanning rate in (mV·s^−1^); m is the mass of the active materials in (g); and t is the time in (s).

(1) $$C=\frac{Q}{V}$$

(2) C=IdVdt x m

The calculated capacitance for graphene oxide using Equation (2) was determined as 180 F·g^−1^, falling within the ranges of 100–200 F·g^−1^ determined by other researchers. Cyclic voltammetry was carried out a number of times in different electrolytes such as Na_2_SO_4_, Li_2_SO_4_ and H_2_SO_4_. The reason for this was to compare and calculate the experimental error. The experimental error was calculated to be very small and this could be due to the experimental conditions, i.e. current density, scan rates and concentration remaining constant.

#### 3.3.2. Electrochemical Impedance Spectroscopy (EIS)

**Figure 9:**
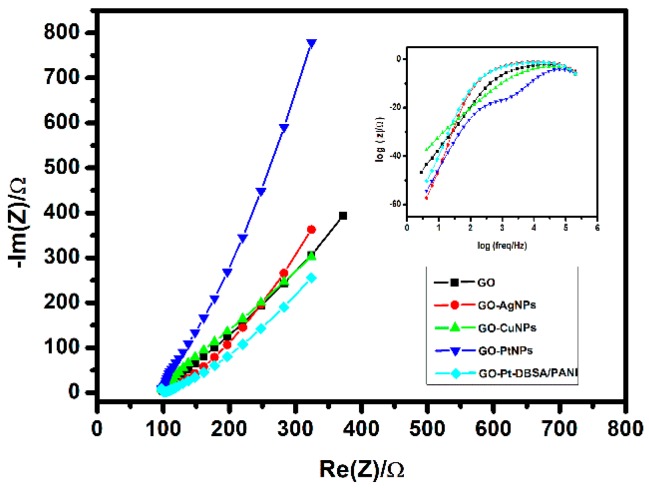
Electrochemical impedance spectroscopy (EIS) Nyquist plot from impedance testing of GO, GO–Ag NPs, GO–Cu NPs, GO–Pt NPs and GO–Pt–DBSA/PANI obtained in 0.1 M Na_2_SO_4_.

Electrochemical impedance spectroscopy (EIS) is an effective method to investigate the interfacial electron transfer characteristics of modified electrodes. The diameter of the semicircles of the Nyquist plots is usually equal to the electron transfer resistance (R_et_) value of 95.45 Ω cm^2^. The relationship between R_et_ and exchange current density (i_0_) is consistent with the equation R_et_ = (RT)/Fi_0_. Here, R is the ideal gas constant (8.314 J mol^−1^ K^−1^) and T is the room temperature (298.15 K). Figure 9 shows the impedance of GO, GO–Ag NPs, GO–Cu NPs, GO–Pt NPs, and GO–Pt–DBSA/PANI, respectively, obtained in aqueous solution of 0.1 M Na_2_SO_4_. The GO showed low resistance compared to the other materials. Moreover, when the GO was modified with the other materials, the resistance increased. These results demonstrate that the nanocomposites effectively enhance the electron transfer efficiency.

### 3.4. Galvanostatic-Charge Discharge Test

**Figure 10:**
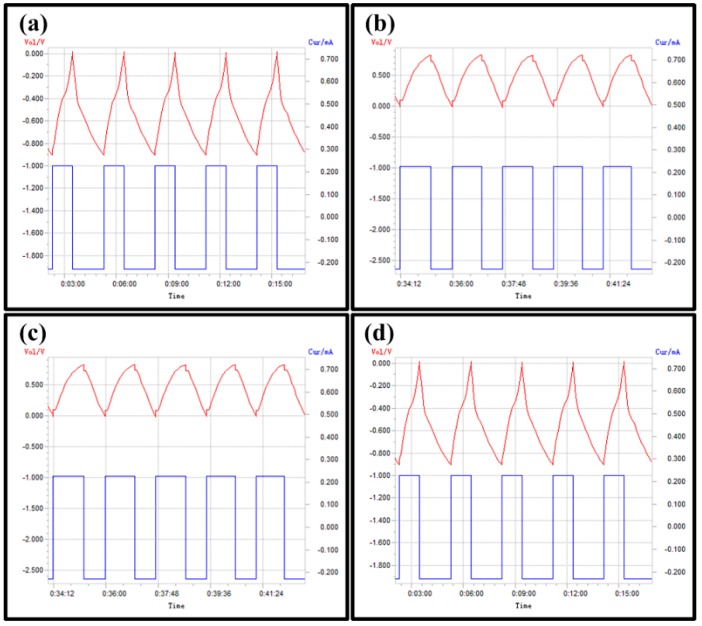
Voltage/time cycling plot of (**a**) GO, (**b**) GO/AgDBSA–PANI (**c**) GO/Cu/DBSA–PANI, (**d**) GO/Pt/DBSA–PANI; voltage range (0 to −0.9 V vs. Ag/AgCl) at 50 A·g^−1^ in 1 M KOH (*aq*). Specific charge/discharge current density = 56.8 A·g^−1^.

The capacitive behaviour of GO, GO/Ag/DBSA–PANI, GO/Cu/DBSA–PANI and GO/Pt/DBSA–PANI is given by the galvanostatic/charge discharge curve as shown in Figure 10a–d respectively, done at a potential range of 0 to −0.9 V. The choice of the potential range is dictated by the choice of the electrolyte. The choice for the electrolyte was aqueous potassium hydroxide (KOH). Its decomposition voltage limit is theoretically 1.23 V, or practically, in kinetic terms, between 1.3 V and 1.4 V. KOH is very soluble in water and because of the OH^−^ anion, it has very good conductivities and advantageously high equivalent conductivities in aqueous medium owing to the special mechanism of proton transport (proton hop-ping) that determines their conductance. The materials showed a large current density in CV curves and a longer charge–discharge time in V-t curves which then implies a larger capacitance. The mass of the thin film of GO was 5.2 g and the first cycle charge and discharge capacities obtained were 44.2 Ah·g^−1^. The voltage range was 0 −0.9 V vs. Ag/AgCl. Cycling was done at a current density of 50 A·g^−1^ in an aqueous solution of 1 M KOH. The specific charge/discharge current density was found to be 45.7 A·g^−1^. The specific capacitance Csp can be calculated, where I is the current density measured in (A), t is the time measured in (s), and V is the voltage scanning rate in (mV·s^−1^):

(3) $$C=\frac{\mathrm{It}}{V}$$

Therefore, specific capacitance, C_sp_, can be calculated: C_sp_ = (45.7 Ag^−1^ × 34.6 s)/0.9 V = 182.8 F·g^−1^. The Csp for GO was calculated as 182.8 F·g^−1^, 206.4 F·g^−1^ for GO/Ag/DBSA–PANI, 192.8 F·g^−1^ for GO/Cu/DBSA–PANI and 227.2 F·g^−1^ for GO/Pt/DBSA–PANI. Comparing these values with the specific capacitance of GO, we can conclude that the materials are good materials and therefore can be used for supercapacitor applications. Platinum is more ductile, is stable at high temperatures and has stable electrical properties when compared to both silver and copper. Hence, platinum has a higher response when compared to the other metals. The experiment was carried out for a number of times under the same conditions which gave reproducible results with non-significant error. Also, coulombic efficiency of the supercapacitor as a function of the number of cycles was performed for 1500 cycles and a retention of up to 96% efficiency was observed. 

## 4. Conclusions

The main aim of this work was to combine the properties of graphene oxide (GO) with the properties of metal nanoparticles (Ag, Cu, Pt) and dodecylbenzene sulfonic acid (DBSA)-doped polyaniline (PANI) to enhance the capacitance, energy and power of developed supercapacitor electrodes. GO has good electrical, mechanical and thermal properties and a high surface area, which makes it a good candidate in applications such as polymer composites and energy-related materials. Metal nanoparticles are exceptionally important due to their unique performance in electronic, magnetic, optical and catalytic applications. Recently, carbon material/conducting polymer/metal oxide nanoparticle composites and carbon material/conducting polymer/metal nanoparticle composites have been used as a new class of composite supercapacitor material with improved properties, when compared to conducting polymer or metal oxide/metal nanoparticle alone. The addition of metal oxides or metal nanoparticles improves the size, morphology and conducting properties of the polymer. GO was successfully synthesized by the modified Hummers method and loaded with Ag, Cu and Pt nanoparticles by the electrostatic self-assembly and finally treated with aniline and DBSA to form conducting polymer composites. Characterization by microscopy techniques, HRTEM and HRSEM, revealed thin, flat, bended, and wrinkled nanosheets, with a size of 1.5 µm, and darker surfaces were shown for the conducting polymer composites. The presence of the functional groups was revealed by EDS. The quantitative analysis of the polymer composites indicates the presence of carbon and sulfur along with silver, copper and platinum for the composites that contain Ag, Cu and Pt. Structural analysis by FTIR confirms the successful loading of the nanoparticles on the surface of graphene oxide and the formation of conducting polymer composites. Prominent bands that confirm the loading of nanoparticles and formation of conducting polymer composites were present. The materials were tested for supercapacitor applications by galvanostatic charge discharge and the specific capacitance of GO was determined as 182.8 F·g^−1^, while for the polymer composites, the specific capacitance was determined as 206.4 for GO/Ag/DBSA–PANI, 192.8 F·g^−1^ for GO/Cu/DBSA–PANI and 227.2 for GO/Pt/DBSA–PANI. There are no reports on graphene oxide loaded with metal nanoparticles combined with DBSA-doped polyaniline for application in supercapacitors, thereby making the use of GO/M/DBSA–PANI a novel concept for the development of high-performance organic supercapacitors.

## Figures and Tables

**Table 1:** Comparison table of specific capacitances, energy and power of different supercapacitor electrode materials over the years.

Material	Specific Capacitance (F·g^−1^)	Specific Energy (Wh·kg^−1^)	Specific Power (W·kg^−1^)	References
GO/Pt/DBSA–PANI	227.2	126.2	178.4	This work
Graphene/MnO_2_//CAN	113.5	51.1	102.2	[33]
TaO_2_–PANI–PSSA	318.4	157.0	435.0	[34]
MnO_2_/e–CMG	389.0	44.0	250.0	[35]
Li_2_MnSiO_4_/Al_2_O_3_	117.5	864.3	104.0	[36]
TiO_2_/CNT	176.5	40.0	6428.0	[37]

**Table 2:** Table showing the wavenumbers and their respective bonds found in GO/Pt NPs and GO/Pt/DBSA–PANI.

GO–Pt NPs		GO/Pt/DBSA–PANI	
Wavenumber (cm^−1^)	Bond	Wavenumber (cm^−1^)	Bond
3436	O-H	3144	N-H
1740	C=O	1590	Quinoid
1636	C=C	1408	Benzenoid
1390	C=C-O	1223	C-N
1220	CO-H	1180	C-H
1040	C-O	1084	O=S=O
-		1004	S-O
